# Editorial

**Published:** 1846-05

**Authors:** 


### THE MEDICAL EXAMINER.

PHILADELPHIA, MAY, 1846.

### THE FRENCH GOVERNMENT AND BICHAT.

By the London Medical Gazette we are informed that: "At the request of the Parisian Medical Congress, Louis Phillipe has ordered that a portrait of Bichat shall be placed in the Historical Gallery of Versailles; and he has conferred a pension on the aged brother of the celebrated anatomist and surgeon."

### THE NATIONAL MEDICAL CONVENTION.

Under this head we regret to find in the last number of the *St. Louis Medical and Surgical Journal,* some exceedingly erroneous and illiberal remarks in reference to the Philadelphia Medical Schools. We shall not quote the offensive observations of our contemporary, nor reply to them, as they were evidently written without much reflection, or a proper acquaintance with the facts; the error, however, into which the writer has fallen, ought to be corrected. It is contained in the following extract: "It seems that though the Convention meets the approbation of most of the Medical Schools and Societies and Journals of the United States, there is some opposition. We regret that this opposition should come from the Philadelphia Schools. Philadelphia has been, and is yet regarded the head quarters of medical teaching in this country, and hence the influence of this city on the destines of the profession, is greater than that which any other city could exert. Should Philadelphia lend her aid in the advancement of Medical reform, this object of the Convention would be much facilitated, but it appears that she will not do this."

We have no special authority to speak for the Philadelphia Schools, but happening to know something of their action in this matter, we venture to assert that the proposed Convention *is not opposed by them.* No such opposition has been manifested or felt. They are entirely passive in regard to it. They have simply come to the conclusion that, *as Medical Schools*, it is not expedient for them to take any part in the Convention; obviously leaving to the profession at large the originating and maturing of any plan or plans that may be thought expedient and right for improving the existing condition of things. Where then is the ground for complaint? Is there any opposition, any illiberality in those who are supposed to have a special and separate interest in the matter, leaving it entirely to the mass of the profession, without seeking in any manner to influence and control its proceedings? We can see none. But why condemn the Philadelphia Schools, as if they alone had declined a participation in the Convention? We have been informed of other schools, and some of no mean pretensions, who have determined to pursue the same course. That some of the Schools should be represented, may be well enough; but if all were to send delegations, so numerous are they that their united voices might control the proceedings of the Convention, and thus give occasion to louder and perhaps well founded complaints.

That there is neither opposition nor indifference felt by the profession in Philadelphia, on this subject, will be seen by the following proceeding of the Philadelphia Medical Society—the oldest and probably the most numerous Medical Association in the United States.

At an adjourned meeting of the Philadelphia Medical Society, held at the Chamber of the College of Physicians, April 22d, 1846, Dr. B. H. Coates, Vice President, in the chair, the following preamble and resolutions were presented by Dr. Warrington:

*Whereas*, a convention of delegates from the different medical societies and colleges of the United States has been called by the New York State Medical Society, to assemble in New York on the first Tuesday in May next; and *whereas*, it is desirable that said convention should be truly national in its character and objects, and should be composed of delegates from the various sections of the Union; therefore,

*Resolved*, That it is expedient for this society to be represented in the proposed meeting.

*Resolved*, That twelve delegates be elected to be present, at the time appointed, to aid in the promotion of such measures and objects as they may deem advisable for the best interests of the medical profession.

On motion of Dr. West, the first resolution was unanimously adopted.

After remarks from several members, the adoption of the second resolution was moved by Dr. Parrish and carried.

On motion, The preamble was unanimously adopted.

On motion of Dr. Wallace, the society proceeded to ballot for each delegate separately, and the Chairman appointed Drs. Bell and Parrish tellers.

The tellers announced the election of the following gentlemen as delegates: Drs. John Bell, Henry Bond, George W. Norris, Isaac Hays,

Isaac Parrish, J. Warringtou, Alfred Stillé, J. Rodman Paul, Francis West, Samuel George Morton, Wm. Pepper and M. Clymer.

On motion of Dr. Bell, it was Resolved, That the delegates have power to fill vacancies in their own body.'

*Resolved,* That the proceedings of this meeting be published in the medical journals, and that the names of the delegates just appointed be published in the daily papers.

Then adjourned.

By order of the Society.

Isaac Parrish, Secretary pro tem.

---

MEDICAL STUDENTS AND GRADUATES.

At our request, a young friend has made out, from official Catalogues and Medical Journals, the following table of the classes in attendance on lectures at the different Medical Colleges the two last years, and of the Graduates, and the ratio of the Graduates to the classes respectively. We shall be glad if some of our cotemporaries will supply the blanks, and correct any errors they may find.

| | CLASS, 1845-46. | GRADUATES, 1846. | RATIO. | RATIO in 1845. |
|---|---|---|---|---|
| Jefferson Medical College, - - - | 469 | 170 | 1 in 2.7 | 1 in 3.5 |
| University of Pennsylvania, - - - | 462 | 168 | 1 in 2.7 | 1 in 2.1 |
| University of New York, - - - - | 425 | 131 | 1 in 3.2 | 1 in 3.2 |
| Medical Institute of Louisville, - - | 345 | 79 | 1 in 4.4 | 1 in 4.0 |
| College of Physicians & Surgeons, N. Y. | 200 | 38 | 1 in 5.3 | 1 in 5.2 |
| Medical College of Ohio, - - - - | 195 | 46 | 1 in 4.2 | 1 in 4.4 |
| Geneva Medical College, - - - - | 178 | | | 1 in 4.4 |
| Harvard University, about - - - | 180 | 31 | 1 in 5.8 | 1 in 8.2 |
| Transylvania University, - - - - | 170 | 64 | 1 in 2.6 | 1 in 4.1 |
| Willoughby Medical College, - - | 164 | | | 1 in 20 |
| Western Reserve College, - - - | 160 | | | |
| Berkshire Medical Institution, - - | 142 | 35 | 1 in 4.1 | |
| Castleton Medical College, - - - | 140 | 36 | 1 in 3.9 | |
| Albany Medical College, - - - - | 115 | 42 | 1 in 2.7 | 1 in 5.5 |
| Medical College of Georgia, - - - | 112 | 31 | 1 in 3.6 | |
| Pennsylvania Medical College, - | | 36 | | 4.3 |
| Medical School of Maine, - - - | 73 | | | |
| Yale College, - - - - - - - - | 53 | | | |
| University of Missouri, - - - - | | 29 | | |
| St. Louis University, - - - - - | | 11 | | |

---

MEDICAL BOTANY.

We have learned with pleasure that a course of lectures on Medical Botany, to commence early in the present month, will be given during the summer by Mr. Alfred L. Kennedy. This will add another to the many facilities afforded in this city to medical students for acquiring a knowledge of medicine and its kindred sciences. We have reason to think Mr. K. is well qualified for the task he has undertaken, and hope our young friends who are remaining in Philadelphia during the summer, will avail themselves of the opportunity for obtaining an acquaintance with this useful and elegant science.

